# QuickStats

**Published:** 2014-04-25

**Authors:** FROM THE NATIONAL CENTER FOR HEALTH STATISTICS

**Figure:**
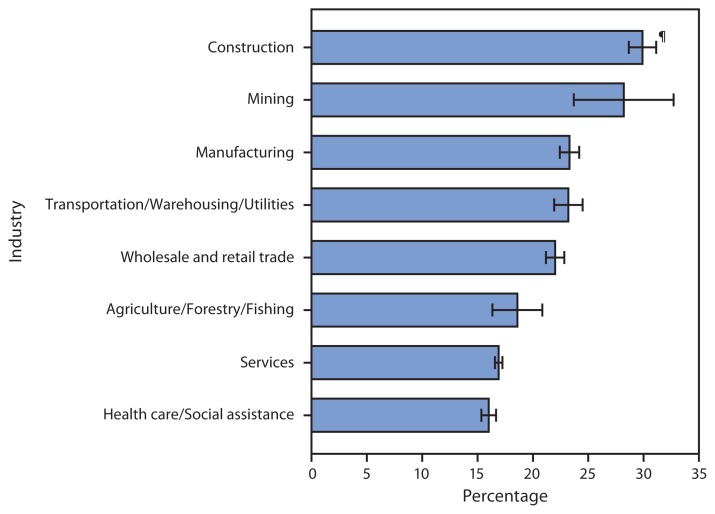
Percentage of Currently Employed Adults Who Were Current Smokers,^*^ by Selected Industries^†^ — National Health Interview Survey, United States, 2008–2012^§^ ^*^ Based on responses to a question that asked, “Have you smoked at least 100 cigarettes in your entire life?” Respondents answering “yes” were then asked, “Do you now smoke cigarettes every day, some days, or not at all?” Current smokers have smoked at least 100 cigarettes in their lifetime and currently smoke every day or some days. ^†^ Industries include the eight sectors emphasized in the National Occupational Research Agenda (http://www.cdc.gov/niosh/nora/sector.html). In the chart above, “Mining” includes oil and gas extraction, and “Services” includes public safety. ^§^ Estimates are based on household interviews of a sample of the U.S. civilian, noninstitutionalized population. Adults who were not currently employed at the time of interview and unknowns with respect to smoking and industry were not included in the denominators when calculating percentages. ^¶^ 95% confidence interval.

During 2008–2012, 29.9% of adults aged ≥18 years currently employed in construction and 28.2% of those currently employed in mining were current smokers. Adults currently employed in construction were more likely than adults currently employed in manufacturing (23.3%), transportation/warehousing/utilities (23.2%), trade (22.0%), agriculture/forestry/fishing (18.6%), services (16.9%), or health care/social assistance (16.0%) to be current smokers.

**Sources:** National Health Interview Survey, 2008–2012. Available at http://www.cdc.gov/nchs/nhis.htm.

**Reported by:** Debra L. Blackwell, PhD, debra.blackwell@cdc.hhs.gov, 301-458-4103.

